# Supplementary material for: HIVprotI: an integrated web based platform for prediction and design of HIV proteins inhibitors
Source: J Cheminform. 2018 Mar 9;10:12. doi: 10.1186/s13321-018-0266-y (PMC5845081; doi:10.1186/s13321-018-0266-y)
Supplement: Supplementary file 2 — Additional file 2. Source code of HIVProtI web server. [file 13321_2018_266_MOESM2_ESM.zip › HIVprotI_Source-code/hivprotI/weka-3-6-6/doc/weka/filters/unsupervised/attribute/PotentialClassIgnorer.html]

PotentialClassIgnorer


---


|  |  |  |
| --- | --- | --- |
| **Overview** \| **Package** \| **Class** \| **Tree** \| **Deprecated** \| **Index** \| **Help** \| **Weka's home** | |  |
| **PREV CLASS**   **NEXT CLASS** | **FRAMES**    **NO FRAMES**     **All Classes** |  |
| SUMMARY: NESTED \| FIELD \| CONSTR \| METHOD | DETAIL: FIELD \| CONSTR \| METHOD |  |


---


## weka.filters.unsupervised.attribute Class PotentialClassIgnorer

```
java.lang.Object
  weka.filters.Filter
      weka.filters.unsupervised.attribute.PotentialClassIgnorer
```

**All Implemented Interfaces:**: java.io.Serializable, CapabilitiesHandler, OptionHandler, RevisionHandler

**Direct Known Subclasses:**: Center, Discretize, MathExpression, Normalize, NumericToBinary, ReplaceMissingValues, Standardize

---

``` public abstract class PotentialClassIgnorer extends Filter implements OptionHandler ```

This filter should be extended by other unsupervised attribute
filters to allow processing of the class attribute if that's
required. It the class is to be ignored it is essential that the
extending filter does not change the position (i.e. index) of the
attribute that is originally the class attribute !

**Version:**
:   $Revision: 1.7 $

**Author:**
:   Eibe Frank (eibe@cs.waikato.ac.nz), Mark Hall (mhall@cs.waikato.ac.nz)

**See Also:**: Serialized Form

---

**Constructor Summary**

`PotentialClassIgnorer()`


| **Method Summary** | |
| --- | --- |
| `boolean` | `getIgnoreClass()`             Gets the IgnoreClass value. |
| `java.lang.String[]` | `getOptions()`             Gets the current settings of the filter. |
| `Instances` | `getOutputFormat()`             Gets the format of the output instances. |
| `java.lang.String` | `ignoreClassTipText()`             Returns the tip text for this property |
| `java.util.Enumeration` | `listOptions()`             Returns an enumeration describing the available options. |
| `void` | `setIgnoreClass(boolean newIgnoreClass)`             Set the IgnoreClass value. |
| `boolean` | `setInputFormat(Instances instanceInfo)`             Sets the format of the input instances. |
| `void` | `setOptions(java.lang.String[] options)`             Parses a list of options for this object. |

**Methods inherited from class weka.filters.Filter**

`batchFilterFile, batchFinished, filterFile, getCapabilities, getCapabilities, getRevision, input, isFirstBatchDone, isNewBatch, isOutputFormatDefined, main, makeCopies, makeCopy, numPendingOutput, output, outputPeek, toString, useFilter, wekaStaticWrapper`

**Methods inherited from class java.lang.Object**

`equals, getClass, hashCode, notify, notifyAll, wait, wait, wait`

**Constructor Detail**

### PotentialClassIgnorer

```
public PotentialClassIgnorer()
```


**Method Detail**

### listOptions

```
public java.util.Enumeration listOptions()
```

:   Returns an enumeration describing the available options.

    :   **Specified by:**: `listOptions` in interface `OptionHandler`
    :   **Returns:**: an enumeration of all the available options.

---


### setOptions

```
public void setOptions(java.lang.String[] options)
                throws java.lang.Exception
```

:   Parses a list of options for this object.

    :   **Specified by:**: `setOptions` in interface `OptionHandler`
    :   **Parameters:**: `options` - the list of options as an array of strings **Throws:**: `java.lang.Exception` - if an option is not supported

---


### getOptions

```
public java.lang.String[] getOptions()
```

:   Gets the current settings of the filter.

    :   **Specified by:**: `getOptions` in interface `OptionHandler`
    :   **Returns:**: an array of strings suitable for passing to setOptions

---


### setInputFormat

```
public boolean setInputFormat(Instances instanceInfo)
                       throws java.lang.Exception
```

:   Sets the format of the input instances. If the filter is able to
    determine the output format before seeing any input instances, it
    does so here. This default implementation clears the output format
    and output queue, and the new batch flag is set. Overriders should
    call `super.setInputFormat(Instances)`

    :   **Overrides:**: `setInputFormat` in class `Filter`
    :   **Parameters:**: `instanceInfo` - an Instances object containing the input instance structure (any instances contained in the object are ignored - only the structure is required). **Returns:**: true if the outputFormat may be collected immediately **Throws:**: `java.lang.Exception` - if the inputFormat can't be set successfully

---


### getOutputFormat

```
public Instances getOutputFormat()
```

:   Gets the format of the output instances. This should only be called
    after input() or batchFinished() has returned true. The relation
    name of the output instances should be changed to reflect the
    action of the filter (eg: add the filter name and options).

    :   **Overrides:**: `getOutputFormat` in class `Filter`
    :   **Returns:**: an Instances object containing the output instance structure only. **Throws:**: `java.lang.NullPointerException` - if no input structure has been defined (or the output format hasn't been determined yet)

---


### ignoreClassTipText

```
public java.lang.String ignoreClassTipText()
```

:   Returns the tip text for this property

    :   **Returns:**: tip text for this property suitable for displaying in the explorer/experimenter gui

---


### setIgnoreClass

```
public void setIgnoreClass(boolean newIgnoreClass)
```

:   Set the IgnoreClass value. Set this to true if the
    class index is to be unset before the filter is applied.

    :   **Parameters:**: `newIgnoreClass` - The new IgnoreClass value.

---


### getIgnoreClass

```
public boolean getIgnoreClass()
```

:   Gets the IgnoreClass value. If this to true then the
    class index is to unset before the filter is applied.

    :   **Returns:**: the current IgnoreClass value.


---


|  |  |  |
| --- | --- | --- |
| **Overview** \| **Package** \| **Class** \| **Tree** \| **Deprecated** \| **Index** \| **Help** \| **Weka's home** | |  |
| **PREV CLASS**   **NEXT CLASS** | **FRAMES**    **NO FRAMES**     **All Classes** |  |
| SUMMARY: NESTED \| FIELD \| CONSTR \| METHOD | DETAIL: FIELD \| CONSTR \| METHOD |  |


---
